# Salmonellosis Outbreak Detected by Automated Spatiotemporal Analysis — New York City, May–June 2019

**DOI:** 10.15585/mmwr.mm6926a2

**Published:** 2020-07-03

**Authors:** Julia Latash, Sharon K. Greene, Faina Stavinsky, Sandy Li, Jennifer A. McConnell, John Novak, Teresa Rozza, Jing Wu, Enoma Omoregie, Lan Li, Eric R. Peterson, Bruce Gutelius, Vasudha Reddy

**Affiliations:** ^1^Bureau of Communicable Disease, New York City Department of Health and Mental Hygiene; ^2^Office of Environmental Investigations, New York City Department of Health and Mental Hygiene; ^3^Public Health Laboratory, New York City Department of Health and Mental Hygiene.

In May 2019, the New York City Department of Health and Mental Hygiene (NYCDOHMH) detected an unusual cluster of five salmonellosis patients via automated spatiotemporal analysis of notifiable diseases using free SaTScan software (*1*). Within 1 day of cluster detection, graduate student interviewers determined that three of the patients had eaten prepared food from the same grocery store (establishment A) located inside the cluster area. NYCDOHMH initiated an investigation to identify additional cases, establish the cause, and provide control recommendations. Overall, 15 New York City (NYC) residents with laboratory-diagnosed salmonellosis who reported eating food from establishment A were identified. The most commonly consumed food item was chicken, reported by 10 patients. All 11 clinical isolates available were serotyped as *Salmonella* Blockley, sequenced, and analyzed by core genome multilocus sequence typing; isolates had a median difference of zero alleles. Environmental assessments revealed food not held at the proper temperature, food not cooled properly, and potential cross-contamination during chicken preparation. Elevated fecal coliform counts were found in two of four ready-to-eat food samples collected from establishment A, and *Bacillus cereus* was detected in three. The outbreak strain of *Salmonella *was isolated from one patient’s leftover chicken. Establishing automated spatiotemporal cluster detection analyses for salmonellosis and other reportable diseases could aid in the detection of geographically focused, community-acquired outbreaks even before laboratory subtyping results become available.

## Investigation and Results

On May 21, 2019, NYCDOHMH detected a spatiotemporal cluster of five salmonellosis patients reported through passive surveillance by electronic laboratory reporting (*2*). These patients resided within a 0.3-mile (0.48-km) radius and had “event dates” (illness onset dates if available, otherwise specimen collection dates) during May 11–17. The cluster’s recurrence interval (*3*) was 2.3 years, indicating that one would expect to see one cluster of that magnitude in any 2.3-year period. This cluster was detected because each weekday, using SaTScan, NYCDOHMH applies the prospective space-time permutation scan statistic (*4*,*5*) to scan for recent increases (parameter settings included maximum temporal cluster size of 60 days and maximum spatial size of 50% of observed events during a 1.5 year-study period) in the occurrence of salmonellosis cases based on patients’ event dates and geocoded home addresses.^†^

At NYCDOHMH, CDC FoodCORE–funded graduate student interns attempt to interview all reported salmonellosis patients as soon as feasible after initial report to collect possible exposure information with minimal recall bias (*6*); median time from report of salmonellosis to completion of interview is generally 2 days. At the time of cluster detection on May 21, interviews had not yet been completed with any cluster patients. The cluster notification prompted interviewers to be vigilant for any common food, grocery store, or restaurant exposures. Once interviews of patients in the initial cluster were completed, student interns immediately compared interviews to look for any common exposures. On May 22, interviewers determined that three of the five patients had eaten prepared food from establishment A.

On May 23, the New York State Department of Agriculture and Markets inspected establishment A to assess food handling practices. On the same day, the NYCDOHMH Office of Environmental Investigations distributed stool collection kits for *Salmonella* testing to 18 food handlers involved in food preparation at establishment A; the first food handler specimen was collected on May 25.

An outbreak-associated case was defined as a laboratory diagnosis of *Salmonella* infection in a NYC resident who reported eating food from establishment A in the 7 days preceding illness onset. Among 17 salmonellosis patients included in the SaTScan cluster during May 21–June 19, interviews were completed with 16 patients, 14 of whom had illnesses meeting the outbreak case definition (Figure). In addition, one food handler not included in the SaTScan cluster also had an illness that met the outbreak case definition but did not cause the outbreak, based on 10 outbreak patients having had symptom onset prior to the food handler. The 15 patients with outbreak-associated cases (14 patrons of establishment A and one food handler) reported eating food from establishment A during May 8–20 and had illness onset during May 14–21. None of the patients resided in the same household. Nine patients were female, and the median age was 42 years (range = 26–61 years). The most common food item consumed, reported by 10 patients, was chicken (rotisserie chicken, chicken salad, or chicken soup). 

**FIGURE:**
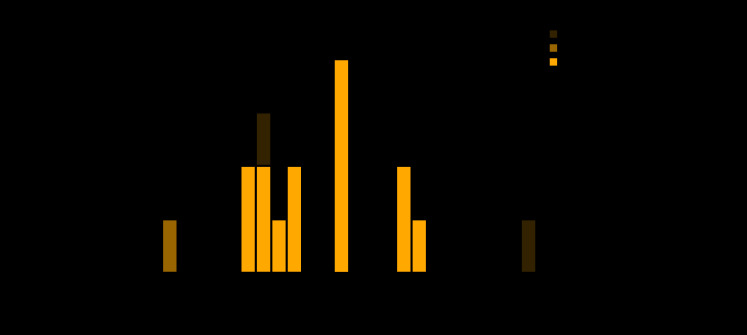
Cases of salmonellosis (N = 17) included in a SaTScan* spatiotemporal cluster, by date reported to health department — New York City, May–June 2019 **Abbreviations:** PFGE = pulsed-field gel electrophoresis; WGS = whole genome sequencing. * https://www.satscan.org/.

Outbreak-associated patient isolates were subtyped at the NYCDOHMH Public Health Laboratory and the New York State Department of Health Wadsworth Center. Eleven of the 15 patients had isolates available for subtyping. All were serotyped as *S*. Blockley, with a median difference by whole genome sequencing (WGS) of zero alleles (range = 0–1 alleles). Pulsed-field gel electrophoresis (PFGE) was performed on nine clinical isolates; all were indistinguishable from each other.

One patient had leftover rotisserie chicken from establishment A, which had not been handled after illness onset and was held under refrigeration until collected by NYCDOHMH on June 1 for testing. *S.* Blockley with an indistinguishable PFGE pattern and 0–1 alleles difference by WGS from the clinical isolates was isolated from the leftover chicken.

On June 5, a second environmental assessment of establishment A was conducted by the New York State Department of Agriculture and Markets jointly with the NYCDOHMH Office of Environmental Investigations. The establishment was immediately notified of violations revealed by this assessment, including ambient temperature of a walk-in refrigerator of 51°F (10.6°C) instead of ≤40°F (4.4°C); opportunities for potential cross-contamination, such as preparing raw chicken in the walk-in cooler and using gloved hands to open walk-in doors during food preparation; using an inadequately calibrated food thermometer; improper hot- and cold-holding of cooked foods; and inadequate cooling of cooked foods. Eight environmental sponge swabs and four ready-to-eat food samples were also collected for testing at NYCDOHMH Public Health Laboratory. The eight environmental swabs tested negative for *Salmonella*, but two ready-to-eat food samples had elevated fecal coliform counts (>1,100 most probable number/gram), and three food samples tested positive for *Bacillus cereus* (range per sample = 70–670 colony-forming units [CFU]/gram), although below the threshold required to cause illness (10^5^–10^6^ CFU/gram) (*7*); these findings were consistent with identified deficiencies in holding temperatures that could allow bacterial proliferation.

To evaluate whether spatiotemporal cluster detection analyses might have contributed to reducing typical delays in taking public health action, the investigation timeline of this outbreak was compared with timelines of previous investigations conducted by NYCDOHMH meeting the following three criteria: 1) the outbreak included at least three patients with a positive laboratory test result for *Salmonella* reported through passive surveillance, such that the outbreak might have been possible to detect via an automated analysis using SaTScan or another method; 2) the investigation occurred during September 2009–May 2019 when graduate student interviewers were in place, such that staffing levels were sufficient to feasibly collect and assess exposures reported by patients (*6*); and 3) the public health response included an environmental assessment of a restaurant or grocery store.

The outbreak described in this report was detected within 2 days of the third case being reported through passive surveillance, compared with a median of 13 days (range = 6–57 days) for five previous outbreaks (Table). An environmental assessment was performed within 2 days of outbreak detection, compared with a median of 21 days (range = 8–111 days) for six previous outbreaks.

**TABLE:** Characteristics of selected* salmonellosis outbreaks — New York City, September 2009–May 2019

Month/Year OB detected	Method by which OB came to attention of NYCDOHMH	Days from third NYC case reported to OB detection	Days from OB detection to first NYC environmental assessment	No. of NYC residents meeting OB case definition	Median age, yrs (range)	% Female	*Salmonella* serotype(s)	Source	Environmental findings
07/2011	Another health dept. notified NYCDOHMH of increase in *S.* Heidelberg among persons in the Orthodox Jewish population	57	111	73	16 (<1–90)	44	Heidelberg	Broiled chicken livers^†^	Chicken livers appeared to be ready-to-eat and were not cooked to appropriate internal temperature; outbreak strain isolated from product samples
02/2012	NYCDOHMH applied historical limits method^§^ to serotyping results	6	21	23	28 (11–74)	57	Bareilly/ Nchanga	Frozen chopped tuna^¶^	Gloves not worn by cooks; product samples collected and tested negative for *Salmonella*
08/2015	PHL identified patient cluster with indistinguishable PFGE patterns	8	21	8	22 (<1–56)	38	Oranienburg	Not determined, but common restaurant	No critical violations noted
03/2018	PHL identified patient cluster with indistinguishable PFGE patterns	22	8	6	28.5 (13–32)	83	Saintpaul	Not determined, but common restaurant	No critical violations noted; product samples collected and tested negative for *Salmonella*
03/2019	Another health dept. notified NYCDOHMH of two NYC cases with PFGE patterns indistinguishable to cases in nearby states	13	48	16	24.5 (3–87)	38	Typhimurium	Not determined, but common restaurant	No critical violations noted
03/2019	NYCDOHMH automated alert for any newly reported *S*. Concord cases following a recent multistate cluster associated with tahini	−30**	21	4	15.5 (<1–30)	50	Concord	Tahini^††^	No critical violations noted; outbreak strain isolated from product samples
05/2019	NYCDOHMH automated spatiotemporal analysis using SaTScan	2	2	15	42 (26–61)	60	Blockley	Chicken at a common grocery store	Improper hot- and cold-holding; potential cross-contamination between raw chicken and ready-to-eat foods; use of poorly calibrated food thermometer; inadequate cooling of cooked foods; elevated fecal coliform counts (two product samples); *Bacillus cereus* detected (three product samples); OB strain isolated from patient’s leftover food

**Abbreviations:** NYC = New York City; NYCDOHMH = New York City Department of Health and Mental Hygiene; OB = outbreak; PFGE = pulsed-field gel electrophoresis; PHL = NYCDOHMH Public Health Laboratory.

* Outbreaks with three or more patients with a positive laboratory test result for *Salmonella* reported through passive surveillance during a period when graduate student interns routinely attempted interviews with all reported salmonellosis patients, and the public health response included an environmental assessment of a restaurant or grocery store.

^†^ Hanson H, et al. Creating student sleuths: how a team of graduate students helped solve an outbreak of *Salmonella* Heidelberg infections associated with kosher broiled chicken livers. J Food Prot 2014;77:1390–3.

^§^ Stroup DF, et al. Evaluation of a method for detecting aberrations in public health surveillance data. Am J Epidemiol 1993;137:373–80.

^¶^ https://www.cdc.gov/salmonella/bareilly-04-12/index.html.

** Ultimately, four patients were included in this cluster, but investigation began after notification of the first patient, given a recently investigated multistate cluster of the same serotype (https://www.cdc.gov/salmonella/concord-11-18/index.html); this cluster was distinct from the previously investigated cluster based on molecular subtyping. This finding was excluded from the summary calculation in the text.

^††^ https://www.cdc.gov/salmonella/concord-05-19/index.html.

## Discussion

This investigation illustrates the utility of integrating automated spatiotemporal cluster detection analyses into applied public health practice. In a jurisdiction with approximately 1,000 salmonellosis cases diagnosed each year,^§^ a focal cluster consisting initially of just five cases was detected by NYCDOHMH before any patient interviews were completed, patient isolates were received at a public health laboratory, or laboratory subtyping results were available. Rapid detection, coupled with interviews conducted by experienced investigators, facilitated food handler testing, environmental assessments highlighting food handling deficiencies, prioritization of patient isolates for molecular subtyping, and collection of patient leftovers for testing before they were discarded. This local investigation, which confirmed chicken as the outbreak source, was later incorporated into a multistate investigation of *S.* Blockley associated with chicken.

It is uncommon for NYCDOHMH to detect a salmonellosis outbreak in the absence of any laboratory subtyping data (*8*) or any single report of multiple ill patients. As of July 15, 2019, CDC PulseNet transitioned its primary molecular subtyping tool from PFGE to WGS, which will improve foodborne outbreak detection through detailed pathogen characterization (*9*,*10*). However, the additional time required for WGS testing could result in a lag in identifying some outbreaks; and some outbreaks might be missed if isolates for subtyping are unavailable with use of culture-independent diagnostic tests, or if not all isolates can be tested, given public health laboratory capacity limitations. In February 2019, ahead of PulseNet’s transition to WGS, NYCDOHMH set up automated analyses using SaTScan to detect salmonellosis clusters without regard to laboratory subtyping results.

Rapid detection of this focal, community-based outbreak relied on critical public health infrastructure and informatics, including automated and timely electronic laboratory reporting, transfer of disease reports to a disease surveillance database, geocoding of patients’ residences, and analyses using SaTScan. Once detected, the rapid outbreak response relied on adequately resourced student interviewers, epidemiologists, environmental health inspectors, and laboratory personnel. Establishing automated spatiotemporal cluster detection analyses for salmonellosis and other reportable diseases could aid in the detection of geographically focused, community-acquired outbreaks.

**Summary**

**What is already known about this topic?**

Whole genome sequencing (WGS) improves detection of foodborne outbreaks caused by contaminated products. However, detecting geographically focal outbreaks can be delayed pending WGS results, and public health laboratory capacity limitations might preclude sequencing of all *Salmonella* isolates.

**What is added by this report?**

Through daily automated spatiotemporal analysis of notifiable diseases, a salmonellosis outbreak in New York City was detected 9 days before availability of serotyping results. Early detection primed investigators to look for common exposures and facilitated rapid environmental assessments, leftover food collection, and prioritization of isolates for subtyping.

**What are the implications for public health practice?**

Along with laboratory subtyping results, public health officials can use spatiotemporal cluster detection analyses to prioritize investigations.

## Footnotes

* These authors contributed equally to this report.

§ https://a816-health.nyc.gov/hdi/epiquery/.
